# Systematics and phylogeny of the entomopathogenic nematobacterial complexes *Steinernema–Xenorhabdus* and *Heterorhabditis*–*Photorhabdus*

**DOI:** 10.1186/s40851-024-00235-y

**Published:** 2024-07-17

**Authors:** Vladimír Půža, Ricardo A. R. Machado

**Affiliations:** 1 Institute of Entomology, Biology centre of the Czech Academy of Sciences, Branišovská 31, České Budějovice, 37005 Czech Republic; 2 Faculty of Agriculture and Technology, University of South Bohemia, Studentská 1668, České Budějovice, 37005 Czech Republic; 3 Experimental Biology Research Group, Institute of Biology, Faculty of Sciences, University of Neuchâtel, Neuchâtel, 2000 Switzerland

**Keywords:** Biological control agents, Beneficial microorganisms, Entomopathogens, Taxonomy, Systematics, Phylogeny

## Abstract

Entomopathogenic nematodes of the genera *Steinernema* and *Heterorhabditis*, along with their bacterial symbionts from the genera *Xenorhabdus* and *Photorhabdus*, respectively, are important biological control agents against agricultural pests. Rapid progress in the development of genomic tools has catalyzed a transformation of the systematics of these organisms, reshaping our understanding of their phylogenetic and cophlylogenetic relationships. In this review, we discuss the major historical events in the taxonomy and systematics of this group of organisms, highlighting the latest advancements in these fields. Additionally, we synthesize information on nematode–bacteria associations and assess the existing evidence regarding their cophylogenetic relationships.

**Supplementary Information:**

The online version contains supplementary material available at 10.1186/s40851-024-00235-y.

## Background

**Fig. 1:**
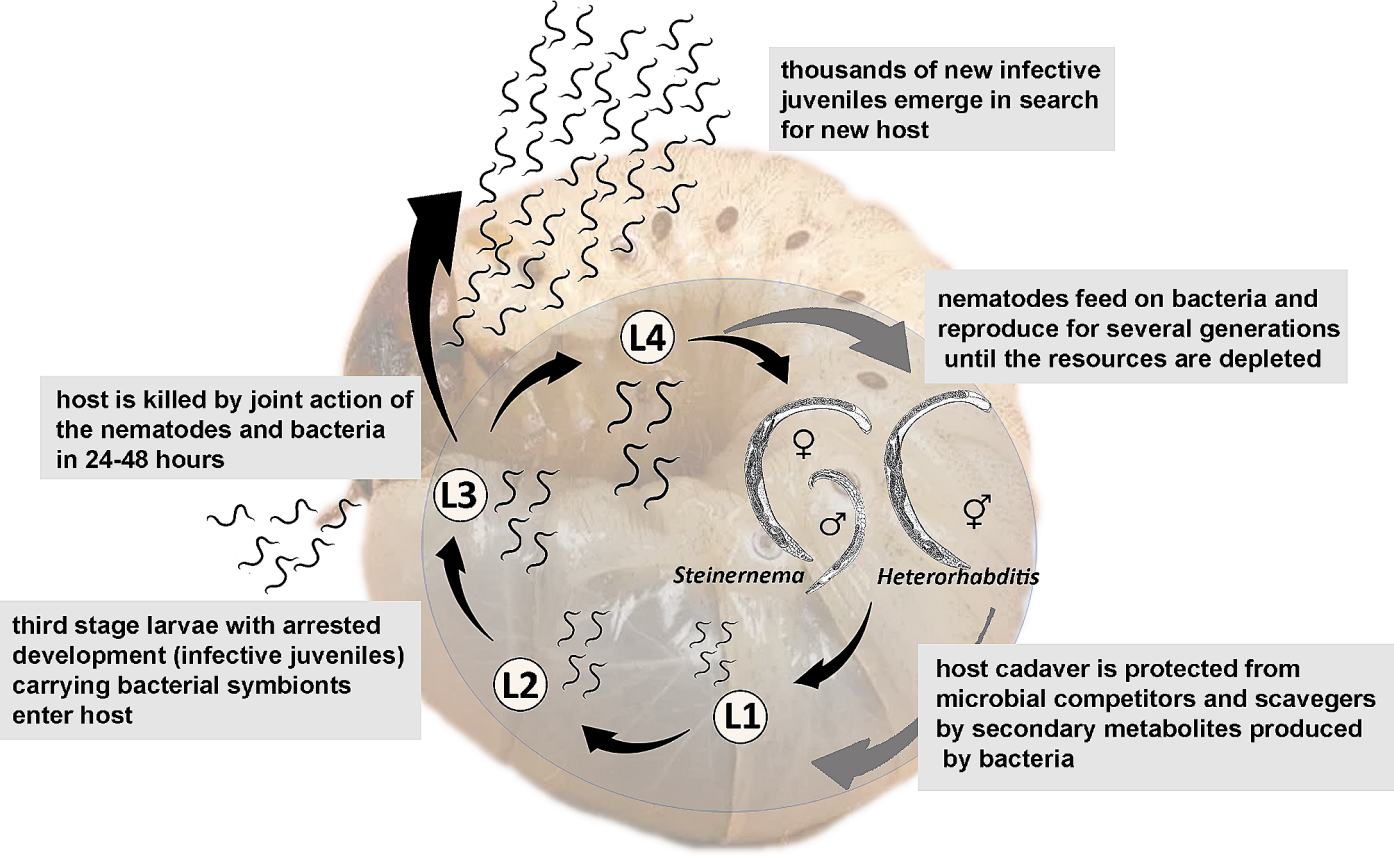
Generalized life cycle of entomopathogenic nematodes

Entomopathogenic nematodes (EPNs) from the genera *Steinernema* and *Heterorhabditis* are obligate lethal pathogens of insects [1]. They establish mutualistic relationships with bacteria of the genera *Xenorhabdus* and *Photorhabdus*, respectively [1–5], and facultative association with several other bacterial species [6–9]. During their life cycle (Fig. 1), the non-feeding third stage nematode larvae (infective juveniles), harbor the mutualistic bacteria in their intestines [10]. These larvae move freely in the soil, and seek insect hosts. Upon locating a suitable insect, the larvae enter its body and release their bacterial symbiont [11]. The bacteria proliferate, and the host is killed typically within 24–48 h by toxins produced by the bacteria and the nematodes [12]. The larvae feed on bacterial cells and develop into amphimictic adults in the genus *Steinernema* or hermaphrodites in *Heterorhabditis* [13]. The first generation is followed by several amphimictic generations in both genera. When the insect carcass is depleted of nutrients, the nematodes revert back to the resting form, and their receptacle is colonized by bacterial cells. This usually occurs within 7–14 days after infection, depending on the host size, temperature, and other factors [14]. Finally, the nematodes re-establish symbiosis with the bacteria and abandon the depleted insect cadaver in search of a new host [15, 16].

Given their effective insect-killing abilities [17], potential for large-scale industrial production [18], coupled with their relative safety towards non-target organisms [19, 20], and environmental considerations, [21] these nematode-bacterial complexes serve as biological control agents and are fundamental pillars of integrated pests management programs [22–24].

The relationship between *Steinernema* and *Xenorhabdus*, and *Heterorhabditis* and *Photorhabdus* is obligate in natural environments [10]. The nematodes transport the bacteria inside soil-borne insects, and through the action of bacterial toxins and digestive enzymes, the infested insect is killed and converted into biomass that is used by the nematodes and the bacteria to proliferate [25–28]. The secondary metabolites produced by the bacterial symbionts also protect the host cadaver against other microorganisms [11, 26, 29, 30] and scavengers [31–33].

The advancement of molecular methods in recent decades has enabled a significant transformation of the systematics of entomopathogenic nematode-bacterial complexes. This review charts the evolution of methods used in the taxonomy of entomopathogenic nematodes and their bacterial symbionts, with a special emphasis on the current state and the latest advances in this field. We also aim to synthesize the published records of nematode–bacteria associations and assess the degree of specificity in *Steinernema–Xenorhabdus* and *Heterorhabditis*–*Photorhabdus* pairs.

## Entomopathogenic nematodes

### Species diversity and species delimitation

The systematics of entomopathogenic nematodes has undergone a revolution with the onset of molecular methods that provide a strong discriminatory tool for morphologically conservative organisms. Consequently, the number of recognized species has significantly increased over 20 years, growing from 22 EPN species in 1995 to 108 in 2015. However, the expansion of molecular methods has also brought certain challenges, including for instance issues related to describing novel species using too short or poorly curated sequences, or using erroneous sequence alignments. Through a comprehensive analysis of available molecular data, the systematics of these nematode groups were revised by Hunt and Subbotin [34], which resulted in the synonymization of more than 10 species, which lacked adequate molecular support, to some previously described species [34]. As of the end of 2023, there are 113 species of *Steinernema* and 21 species of *Heterorhabditis* [35], and several new EPN species descriptions are expected to be published in the near future.

Approaches for the delimitation of EPN species have long been a matter of discussion. Adams [36], for instance, argued that the traditionally used approach based on overall molecular/morphological similarities or reproductive compatibility neglects historical relationships and likely fails to accurately reflect the number of actually existing species. He proposed to delimitate species based on unique character states to show evidence for lineage independence. Spiridonov et al. [37] argued that with the increasing number of known species, autapomorphies for some well-established species may disappear, and suggested that sequence divergence is a better indication of lineage independence. Adams et al. [38] however disagreed with this view and suggested that sequence divergence cannot reveal lineage independence and considered its use to delimit species to be arbitrary, and thus a poor indicator of species boundaries. At present, EPN species are delimited using an amalgamation of evolutionary and phylogenetic species concepts [36] while sequence divergence is currently the primary method for identifying EPN species.

The dominant molecular marker used in EPN systematics is the sequence of the internal transcribed spacer regions of the rDNA tandem repeat unit (ITS1–5.8 S–ITS2). In the case of *Steinernema* species, this gene marker is suitable to resolve the relationships among closely related species, and it is the most widely used marker for the diagnosis and identification of new EPN species [39]. Nguyen [40] noted that a 5% sequence divergence could effectively distinguish between *Steinernema* species present at the time. However, subsequently described closely related species exhibit variances of no more than 3% in the sequences of the ITS region [41]. In certain *Steinernema* species, the use of ITS sequence can be complicated by intra-individual variability in the ITS sequences of some *Steinernema* species [39]. Gene markers, such as the D2D3 expansion segment of the LSU rDNA sequence, are compulsory in description of EPN species due to their use in metabarcoding studies, but the segment is too conserved to distinguish between closely related species. Some recent studies describing novel steinernematid species are also reporting the sequences of the mitochondrial 12 S rRNA and of the cytochrome oxidase subunit I (COI) (e.g. [42]), anticipating that ITS and D2D3 sequences might later provide insufficient information as the number of novel species is rapidly increasing.

In the genus *Heterorhabditis*, that is evolutionarily younger compared to *Steinernema* [38], there is a lower variability in the standardly used markers (especially the D2D3, but also the ITS regions of the rDNA). Recently, Dhakal et al. [43] analyzed COI, unc-87 encoding thin filament (F-actin)-associated protein and cmd-1 gene encoding calmodulin of a large number of *Heterorhabditis* species and isolates. As a result, the analyses confirmed the synonymization of several species suggested by Hunt and Subbotin [34], and revealed the possibility that some isolates might have been misidentified and actually represent different, undescribed species. Indeed, three new species, namely *H. ruandica* and *H. zacatecana* [44] and *H. casmirica* [45] were recently described using a multilocus approach, as the D2D3 sequences were identical in some cases, and the ITS sequences nearly identical to the sequences of the closely related species *H. bacteriophora.* This highlights the necessity of transitioning to multilocus molecular characterization, or even to the use of core genome sequences for future EPN systematics and species descriptions.

### Phylogeny

Although steinernematid and heterorhabditid nematodes exhibit many similarities in their life histories, they are representatives of two different evolutionary lineages within the Rhabditida order. According to Poinar 2011, both families are of Permian origin (230–252 mil. years). Based on morphological similarities and molecular data, the family Heterorhabditidae was considered as a member of the superfamily Strongyloidea, and the family Steinernematidae of the superfamily Strongyloididea [46]. Based on recent single and multi-locus analyses, the family Heterorhabditidae indeed forms a basal group of Strongyloidea [47–49] whereas recent phylogenomic analysis have shown the family Steinernematidae as the earliest branching clade of the group Tylenchina [49].

Nguyen et al. [50] were the first to propose dividing the genus *Heterorhabditis* into three clades (groups): ‘*Indica*’, ‘*Bacteriophora*’ and ‘*Megidis*’ and this division was confirmed by other authors [43, 51]. The “*Indica*” clade, named after pantropical species *H. indica*, contains seven species that predominantly occur in tropics and subtropics. This clade is an outgroup to the ‘*Bacteriophora*’ and ‘*Megidis*’ clades. The “*Bacteriophora*” clade contains the most widespread species, *H. bacteriophora*, and five other species with a very narrow geographic range. The ‘*Megidis*’ clade includes six species, among them *H. megidis*, with a Holarctic distribution and *H. zealandica*, which occurs in several continents from both hemispheres. While some relationships within the clades are well-supported, others are not resolved with currently available molecular markers. Generally, well-supported relationships between the clades are only provided by using ITS rRNA gene sequences [43].

The first comprehensive phylogenetic analysis of the family Steinernematidae based on the sequence of D2-D3 expansion segments of the 28S rDNA gene revealed five main clades within the family Steinernematidae [52]. The following analysis based on the ITS rDNA sequence made by Spiridonov et al. [37] divided the family into 5 main clades (clade I: *affine*-*intermedium*; clade II: *carpocapsae*-*scapterisci-tami*; clade III: *feltiae*-*kraussei*-*oregonense*, clade IV *bicornutum*-ceratophorum-*riobrave* and clade V: *arenarium-glaseri*-*karii*-*longicaudum*. The latest comprehensive analysis [51] divided the group into twelve multiple species/clades: “Affine”, “Bicornutum”, “Cameroonense”, “Carpocapsae”, “Costaricense”, “Feltiae”, “Glaseri”, “Karii”, “ Khoisanae”, “Kushidai”, “Longicaudum” and, “Monticola”; and three monospecies clades: *S. neocurtillae*, *S. unicornum*, and *S. rarum*. In a similar manner as for the Heterorhabditidae family, currently available molecular markers are insufficient to clarify the relationships within some of the larger clades. To clarify currently unresolved relationships, future phylogenetic studies could prioritize finding additional genetic markers. Alternatively, they could focus on conducting phylogenomic analyses.

## Bacterial symbionts

### The origins of the bacterial genera *Photorhabdus* and *Xenorhabdus*

**Fig. 2:**
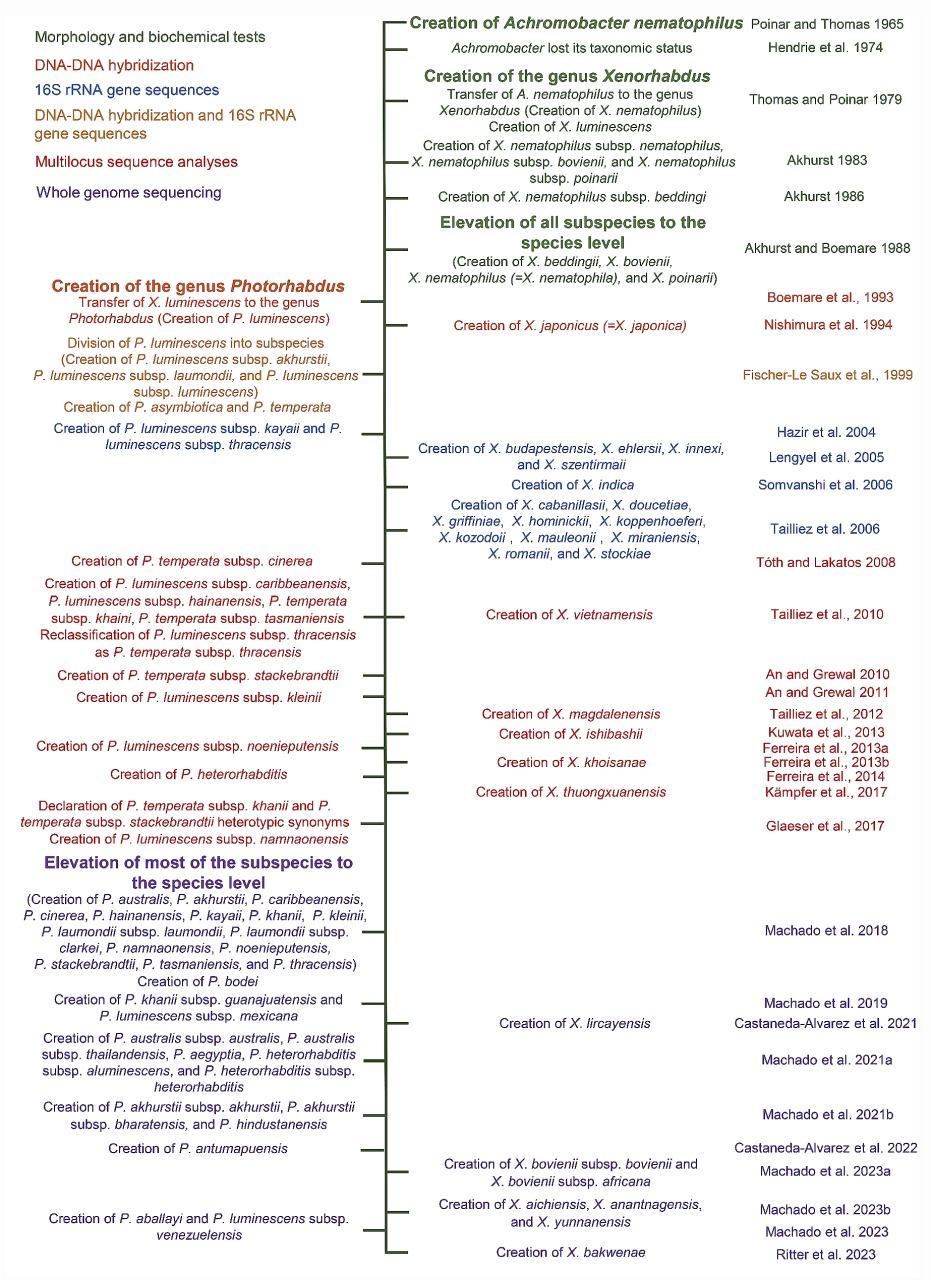
Major events in the taxonomic history of the symbiotic bacteria associated to *Steinernema* and *Heterorhabditis* nematodes, currently classified within the genera *Xenorhabdus* and *Photorhabdus*, respectively

The first taxonomic study of symbiotic bacteria associated with entomopathogenic nematodes was carried out to characterize a bacterial species isolated from the intestinal lumen of *Neoaplectana carpocapsae* Weiser (Steinernematidae: Nematoda; Syn: *Steinernema carpocapsae*) [53–56] (Fig. 2). This bacterial species was named *Achromobacter nematophilus* Poinar and Thomas 1965 [54] based on morphological characters and biochemical traits. Several subsequent studies were conducted to describe the biology of this bacterial species, including its entomopathogenic abilities [54, 57–61]. A few years later, the genus *Achromobacter* lost its status and several of its species were transferred to the genus *Alcaligenes* Castellani and Chalmers 1919 [62, 63], leaving the species *Achromobacter nematophilus* in a taxonomic limbo.

The bacteria associated to entomopathogenic nematodes continued to raise scientific interests and additional strains were isolated and characterized [58–61]. During the characterization of several bacterial strains isolated from *Heterorhabditis bacteriophora* and *Neoplectana* (= *Steinernema*) nematodes, Gerard M. Thomas and George O. Poinar Jr. noticed that the strains isolated from *H. bacteriophora* nematodes shared several characteristics with strains isolated from *Neoplectana* nematodes, including the type strain of the species *A. nematophilus*, but differed in bioluminescence production and catalase activity [64]. Consequently, they proposed: (i) the creation of the genus *Xenorhabdus* Thomas and Poinar 1979 to accommodate large, gram-negative, rod-shaped, facultatively anaerobic, entomopathogenic bacteria which are intimately associated with entomopathogenic nematodes; (ii) to transfer *A. nematophilus* to this new genus, hence the creation of *X. nematophilus*, and (iii) the creation of a novel species, *X. luminescens* to accommodate the bioluminescent strains isolated from *Heterorhabditis* nematodes [64]. Noteworthy to mention that the correct spelling of the species *X. nematophilus* is *X. nematophila*, to conform to the grammar rules of the Latin language. Correct spelling was introduced in the literature from 2000 [65, 66]. To avoid confusion, we will use the scientific names as they were originally proposed. The collection of *Xenorhabdus* strains rapidly increased, allowing deeper characterization of the bacterial genus, which served as grounds for establishing the multispecies nature of the genus and to describe novel taxa [67–77]. Taxonomists rapidly realized that relying merely on morphological and biochemical characters was not sufficient to confidently discriminate the different taxa, hence, several by-then state-of-the-art techniques started to be included in studies, such as DNA-DNA hybridization, 16 S sequences, and fatty acid methyl ester (FAME) profiling [71, 77–82].

The results of these studies often provided evidence for the phenotypic and genetic divergence between species, but also showed that all the available strains can be grouped into two distinct groups: one composed of strains that produce bioluminescence and are associated with *Heterorhabditis* nematodes, and a second group composed of strains that are aluminescent and are associated with *Neoaplectana* (Syn: *Steinernema*) nematodes [83]. Consequently, Noël Boemare, Raymond Akhurst, and Roslyn Mourant carried out a large study including several strains, and based on DNA-DNA hybridization studies proposed the creation of a novel bacterial genus, *Photorhabdus*, transferring thereby those bioluminescent strains associated with *Heterorhabditis* nematodes [2]. Several further studies provided evidence, often genetic evidence, of the distinctiveness of these two genera [84–91].

### History of the taxonomy of the genus *Xenorhabdus*

The first described species of the genus *Xenorhabdus* was *X. nematophilus*, which resulted from the proposal to transfer *A. nematophilus* to this newly created genus [64](Fig. 2). Shortly after its creation, the species *X. nematophilus* was divided into three subspecies: *X. nematophilus* subsp. *nematophilus*, *X. nematophilus* subsp. *bovienii*, and *X. nematophilus* subsp. *poinarii* [83]. Subsequently, the creation of *X. nematophilus* subsp. *beddingii* was proposed [73]. Using a numerical approach based on biochemical characteristics, all *X. nematophilus* subspecies were proposed to be elevated to the species status, which led to the creation of the following species: *X. beddingii*, *X. bovienii*, *X. nematophilus*, and *X. poinarii* [75]. A few years later, the use of 16 S rRNA gene sequences for taxonomic purposes became standard and boosted the discovery of novel species of the genus, increasing the number of *Xenorhabdus* species to twenty [77, 92–94]. Since 2006, the description of novel species was somewhat slow, but the multi-locus sequence analysis (MLSA) approach was implemented, increasing the robustness of the taxonomic conclusions derived from such studies [95–99]. Since 2021, the use of core genome sequences became the norm to describe novel species [100–103]. Using this approach, the proposal for dividing a *Xenorhabdus* species, *X. bovienii*, into two subspecies, *X. bovienii* subsp. *bovienii* and *X. bovienii* subsp. *africana*, was made for the first time [102](Fig. 2).

### History of the taxonomy of the genus *Photorhabdus*

The bacterial genus *Photorhabdus* was established by Boemare et al. [2] to harmonize the taxonomy of bacteria symbiotically associated with entomopathogenic nematodes (Fig. 2). Initially, this genus contained a single species, *P. luminescens* [2]. Several further taxonomic studies were carried out, which provided evidence to suggest that *P. luminescens* was actually a heterogeneous genomic group, likely composed of several distinct species [84, 86–89, 104]. Decisive evidence for this notion was first provided by Fischer-Le Saux et al. [90], who measured DNA relatedness levels across several *Photorhabdus* strains. Consequently, they proposed to create two new species, *P. asymbiotica* and *P. temperata*, and to divide *P. luminescens* into different subspecies: *P. luminescens* subsp. *akhurstii*, *P. luminescens* subsp. *laumondii*, and *P. luminescens* subsp. *luminescens* [90]. Importantly, the arguments to support the novel species and subspecies relied on an 80% DNA relatedness threshold proposed by Vandamme et al. [105] instead of the 70% threshold proposed by the ad hoc Committee on Reconciliation of Approaches to Bacterial Systematics [105, 106]. The following studies describing novel *Photorhabdus* taxa assigned them the status of subspecies, despite the fact that DNA relatedness scores supported their status as species, perhaps to preserve the more conservative subspecies system proposed by Fischer-Le Saux et al. [90]: [90, 95, 107–112]. Hence the bacterial species concept in the *Photorhabdus* genus was initially outlined as a collection of strains that share at least one diagnostic phenotypic trait and whose purified DNA molecules show at least 80% cross-hybridization. With the rapid advances in DNA sequencing technology, additional quantitative phylogenetic methods were developed to replace the wet-lab DNA-DNA hybridization method, as multi-locus sequence analysis (MLSA) [99, 112, 113]. Despite the clear phylogenetic power of the MLSA approach, the taxonomy of the genus *Photorhabdus* was not totally clear, as major taxonomic uncertainties were evident due to the use of a 97% nucleotide sequence identity (NSI) cutoff to delimit subspecies boundaries instead of species boundaries, as it was commonly used in many other bacterial groups [95, 111, 114–116]. As a consequence, very closely related species such as *P. temperata* subsp. *khanii* and *P. temperata* subsp. *stackebrandtii*, which share 98.4% nucleotide sequence similarity of concatenated housekeeping genes between them, were declared heterotypic synonyms [95, 109, 113]. In addition, the application of the 97% threshold resulted also in the misclassification of other isolates. Strains KR04 and C8406, for instance, were initially classified as *P. luminescens* subsp. *kayaii* in spite of their phylogenetic separation from strains FR33 and CIP 108,428^T^, both of them classified as *P. luminescens* subsp. *kayaii* using MLSA [95, 111]. Similar taxonomic misplacements were observed in other taxa such as *P. luminescens* subsp. *laumondii*, *P. luminescens* subsp. *kayaii* and *P. luminescens* subsp. *kleinii* [110].

To harmonize the taxonomy of the genus *Photorhabdus*, Machado et al. [117] implemented whole-genome-based approaches that were becoming the gold standard for bacterial taxonomy at that time [117–123]. Using sequence comparison approaches such as orthologous average nucleotide identity (OrthoANI) and in silico DNA-DNA hybridization (isDDH) and by reconstructing phylogenetic relationships based on core genomes, Machado et al. [117] proposed the elevation of most of the subspecies of the genus *Photorhabdus* to the species level [117]. Since then, the use of whole genome-based approaches has become the norm, and several novel species and subspecies have been proposed using this approach [44, 117, 124–127](Fig. 2).

### Current taxonomic status and current standards to describe *Xenorhabdus* and *Photorhabdus* species

**Fig. 3:**
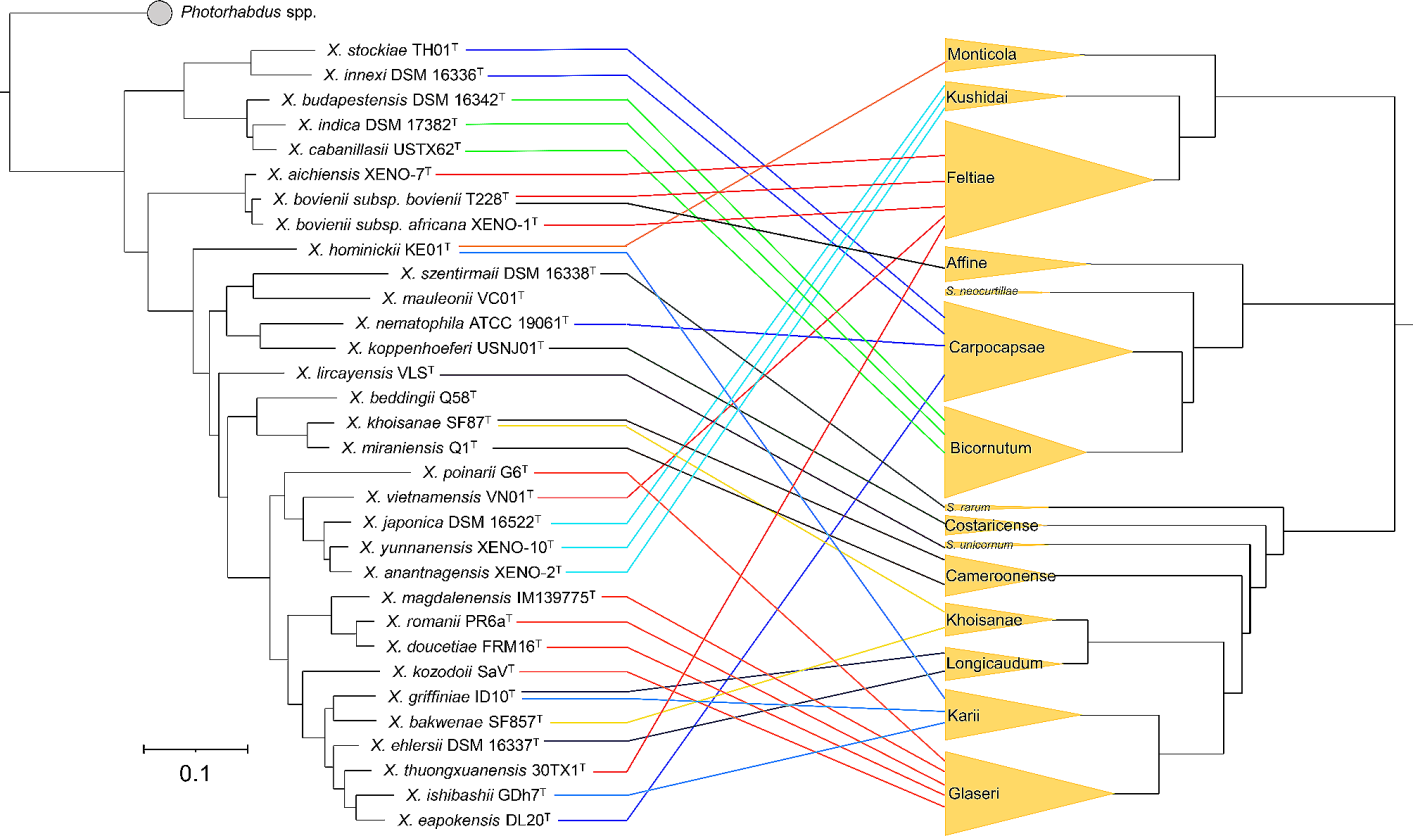
Phylogenetic trees of *Xenorhabdus* bacteria and main steinernematid clades and their associations. Phylogenetic relationships among *Xenorhabdus* were reconstructed based on core genome sequences of *Xenorhabdus* type strains. 1,466,520 nucleotide positions (1439 core genes) were used in the analyses. Bar represents 0.05 nucleotide substitutions per sequence position. NCBI accession numbers of the genome sequences used for the reconstruction are shown in Table S1. Phylogenetic relationships within *Steinernema* clades are based on Spirodonov and Subbotin [51]. The associations between nematodes and bacteria are depicted by lines, and different line colors distinguish the associations of nematodes from distinct clades

**Fig. 4:**
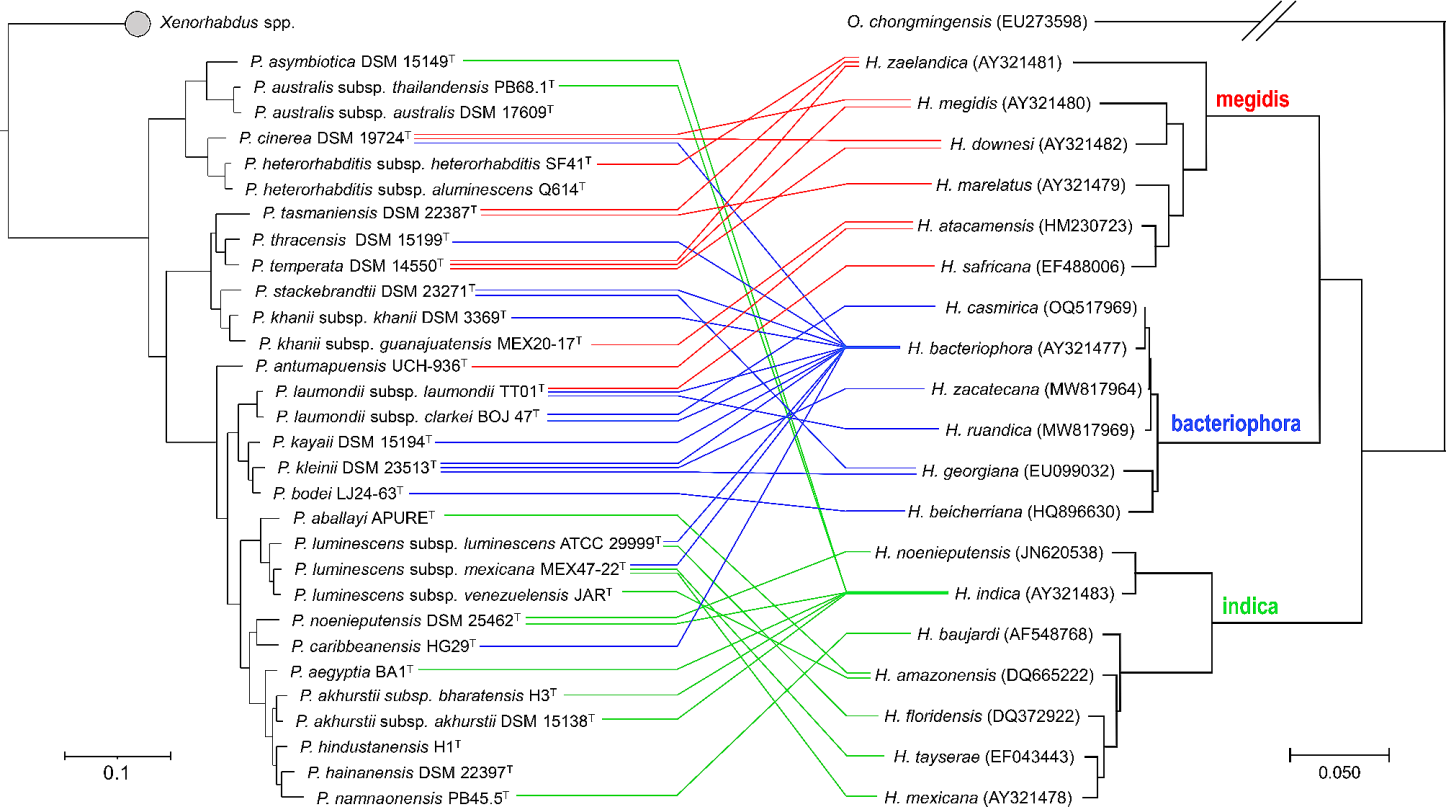
Phylogenetic trees of *Photorhabdus* bacteria and *Heterorhabditis* nematodes and their associations. Phylogenetic relationships among *Photorhabdus* were reconstructed based on core genome sequences of *Photorhabdus* type strains with validly published names. 2,236,770 nucleotide positions (2231 core genes) were used in the analyses. Bar represents 0.05 nucleotide substitutions per sequence position. NCBI accession numbers of the genome sequences used for the reconstruction are shown in Table S2. Phylogenetic relationships within *Heterorhabditis* species were inferred by Minimum Evolution analysis of the ITS rDNA gene. The associations between nematodes and bacteria are depicted by lines, and different line colors distinguish the associations of nematodes from distinct clades

As described above, the description of bacterial species of the genera *Xenorhabdus* and *Photorhabdus* was initially based on morphological and biochemical differences, followed by DNA-DNA hybridization assays, and then by genetic differences of few genetic markers, such as the 16 S rRNA gene, the recombinase A (*recA*), the DNA polymerase III beta subunit (*dnaN*), the glutamyl-tRNA synthetase (*gltX*), the gyrase beta subunit (*gyrB*), and the translation initiation factor IF-2 (*infB*) [2, 90, 95](Fig. 2). In 2018 and 2021, the use of whole-genome sequences for taxonomic purposes was introduced for the genera *Photorhabdus* and *Xenorhabdus*, respectively, to align with the gold standards for bacterial taxonomy at that time [117, 126]. Now, novel bacterial species are described based on a well-supported phylogenomic separation as phylogenomic trees often capture intra- and interspecific variability, and based on overall genomic relatedness indices (OGRIs) such as average nucleotide identity (ANI) and digital DNA-DNA hybridization (dDDH). Phylogenomic reconstructions are carried out based on core-genome sequences using tools such as Roary and FastTree [128, 129], and overall genomic relatedness indices (OGRIs) are calculated using tools such as the orthologous average nucleotide identity (OrthoANI) and the Genome-to-Genome Distance Calculator in the case of ANI and dDDH, respectively. There are plenty of user-friendly, free, online platforms that carry out these analyses in a fully automated manner, such as The Type (Strain) Genome Server (TYGS) [130, 131]. There are several additional resources for this purpose [132]. A great consensus on the use of OGRIs and on the thresholds that delimit prokaryotic species and subspecies boundaries has now been reached [118, 121, 133, 134]. However, the proposed thresholds values are not fixed values and should be analyzed in a genus-specific manner. In the case of *Photorhabdus* and *Xenorhabdus*, in general terms, two strains belong to different species/subspecies if the dDDH value between them is lower than 70% and/or the ANI value between them is lower than 95–96%. Two strains belong to the same species but different subspecies if the dDDH value is between 70 and 79% and/or the ANI value is between 96 and 98%, and two strains belong to the same species and the same subspecies if their dDDH value is greater than 79% and/or the ANI value is greater than 98%. Based on these values and phylogenomic separations, the bacterial genus *Xenorhabdus* is divided into 32 taxa (31 species, one of which is divided into two subspecies) and the bacterial genus *Photorhabdus* in 30 taxa (23 species, six of which are divided into different subspecies) (Tables 1 and 2; Figs. 3 and 4).

**Table 1:** List of valid *Steinernema* species and *Xenorhabdus* species and subspecies and information on their associations

Nematode	Clade	Bacterium	Nematode	Clade	Bacterium
*S. affine*	affine	X. bovienii* [75]	*S. apuliae*	glaseri	*X. kozodoii* [94]
*S. arasbaranense*		-	*S. arenarium*		*X. kozodoii* [94]
*S. beddingi*		-	*S. australe*		*X. magdalenensis* [96]
*S. intermedium*		*Xenorhabdus* sp. close to *X. bovienii* [89, 102]	*S. boemarei*		*X. kozodoii* [135]
*S. poinari*		X. bovienii* [136]	*S. brazilense*		*-*
*S. sichuanense*		X. bovienii* [94]	*S. caudatum*		*-*
*S. thesami*		X. bovienii* [137]	*S. cubanum*		*X. poinarii* [138]
*S. abbasi*	bicornutum	*X. indica* [93]	*S. diaprepesi*		*X. doucetiae* [94]
*S. bicornutum*		*X. budapestensis* [92]	*S. glaseri*		*X. poinarii* [138]
*S. biddulphi*		*Xenorhabdus* sp. close to *X. indica* [139]	*S. khuongi*		*-*
*S. bifurcatum*		*Xenorhabdus* sp. close to *X. indica* [140]	*S. phyllophagae*		*-*
*S. ceratophorum*		*X. budapestensis* [94]	*S. puertoricense*		*X. romanii* [94]
*S. goweni*		-	*S. riojaense*		*X. kozodoii* [141]
*S. kandii*		*X. indica* [142]	*S. vulcanicum*		*X. kozodoii* [143]
*S. mitclani*		-	*S. aciari*	karii	*X. ishibashii* [98]
*S. pakistanense*		*X. indica* [144]	*S. ethiopiense*		*-*
*S. papillatum*		-	*S. indicum*		*X. griffiniae* [145]
*S. ralatorei*		-	*S. karii*		*X. hominickii* [94]
*S. riobrave*		*X. cabanillasii* [94]	*S. leizhouense*		*-*
*S. shori*		*Xenorhabdus* sp. close to *X. indica* [146]	*S. litchii*		*X. griffiniae* [147]
*S. yirgalemense*		*X. indica* [148]	*S. loci*		-
*S. beitlechemi*	cameroonense	*X. khoisanae* [149]	*S. pwaniensis*		*Xenorhabdus* sp. close to *X. griffiniae* and *X. ehlersii* [150]
*S. bertusi*		-	*S. thanhi*		-
*S. cameroonense*		*Xenorhabdus* sp. close to *X. miraniense* [151]	*S. bakwenae*	khoisanae	*X. bakwenae* [103]
*S. fabii*		*X. khoisanae* [152]	*S. innovationi*		*-*
*S. nyetense*		-	*S. jeffreyense*		*X. khoisanae* [153]
*S. sacchari*		*X. khoisanae* [153]	*S. khoisanae*		*X. khoisanae* [97]
*S. asiaticum*	carpocapsae	-	*S. tophus*		*-*
*S. backanense*		-	*S. akhursti*	kushidai	*X. yunnanensis* [101]
*S. balochiense*		-	*S. anantnagense*		*X. anantnagensis* [101]
*S. carpocapsae*		*X. nematophila* [83]	*S. kushidai*		*X. japonica* [77]
*S. colombiense*		-	*S. populi*		*-*
*S. cumgarense*		-	*S. guangdongense*	longicaudum	*-*
*S. eapokense*		*X. eapokensis* [99]	*S. hermaphroditum*		*X. griffiniae* [94]
*S. huense*		*Xenorhabdus* sp. close to *X. stockiae* [154]	*S. lamjungense*		*-*
*S. minutum*		*X. stockiae* [155]	*S. longicaudum*		*X. ehlersii* [92]
*S. nepalense*		-	*S. pui*		*-*
*S. ritteri*		-	*S. taiwanensis*		*-*
*S. sasonense*		-	*S. ashiuense*	monticola	*X. hominicki* [156]
*S. scapterisci*		*X. innexi* [92]	*S. borjomiense*		*-*
*S. siamkayai*		*X. stockiae* [94]	*S. changbaiense*		*-*
*S. surkhetense*		*Xenorhabdus* sp. close to *X. stockiae* [157]	*S. monticolum*		*X. hominickii* [94]
*S. tami*		-	*S. robustispiculum*		*-*
*S. costaricense*	costaricense	close to *X. koppenhoeferi* and *X. khoisanae* [158]	*S. schliemanni*		close to *X. hominickii* [159]
*S. scarabaei*		*X. koppenhoeferi* [94]	*S. neocurtillae*	-	-
*S. africanum*	feltiae	*X. bovienii* subsp. *africana* [160]	*S. rarum*	-	*X. szentirmaii* [92]
*S. citrae*		X. bovienii* [152]	*S. unicornum*	-	*X. lircayensis* [100]
*S. feltiae*		X. bovienii* [75]	*Steinernema* sp.		*X. beddingii* [94]
*S. hebeiense*		*-*	*Steinernema* sp.		*X. mauleonii* [94]
*S. cholashanense*		*Xenorhabdus* sp. close to X. bovienii**	Steinernematidae		*X. miraniensis* [94]
*S. ichnusae*		X. bovienii* [161]			
*S. jollieti*		*Xenorhabdus* sp. close to X. bovienii* [102, 162]			
*S. kraussei*		X. bovienii* [89]			
*S. litorale*		*X. aichiensis* [101]			
*S. nguyeni*		X. bovienii* [153]			
*S. oregonense*		*Xenorhabdus* sp. close to X. bovienii* [102, 163]			
*S. puntauvense*		*X. bovienii* subsp. *bovienii* [102, 163]			
*S. sandneri*		-			
*S. sangi*		*X. thuongxuanensis* [99] and *X. vietnamensis* [95]			
*S. silvaticum*		X. bovienii* [164]			
*S. texanum*		*-*			
*S. tielingense*		X. bovienii* [165]			
*S. weiseri*		*X. bovienii* subsp. *bovienii* [94, 102]			
*S. xinbinense*		X. aichiensis**			
*S. xueshanense*		*Xenorhabdus* sp. close to X. bovienii**			

* With the current data, it is not possible to determine the exact taxonomic identity as *X. bovienii* subsp. *bovienii* and *X. bovienii* subsp. *africana* are genetically very similar. Whole genome sequences are required in these cases

** this study

**Table 2:** List of valid *Heterorhabditis* species and *Photorhabbdus* species and subspecies and the information on their associations

Nematode	Clade	Bacterium
*H. bacteriophora*	bacteriophora	*P. caribbeanensis, P. cinerea, P. thracensis, P. kleinii, P. khanii* subsp. *khanii, P. laumondii* subsp. *laumondii, P. stackebrandtii, P. laumondii* subsp. *clarkei, P. kayaii, P. luminescens* subsp. *mexicana* [90, 117]
*H. beicherriana*		*P. bodei* [117]
*H. casmirica*		*P. laumondii* subsp. *clarkei* [45]
*H. georgiana*		*P. stackebrandtii, P. kleinii* [117]
*H. ruandica*		*P. laumondii* subsp. *laumondii* [44]
*H. zacatecana*		*P. kleinii* [44]
*H. amazonensis*	indica	*P. aballayi, P. luminescens* subsp. *venezuelensis* [127]
*H. baujardi*		*P. namnaonensis* [117]
*H. floridensis*		*P. luminescens* subsp. *luminescens* [166]
*H. indica*		*P. akhurstii* subsp. *akhurstii, P. akhurstii* subsp. *bharatensis, P. asymbiotica, P. australis* subsp. *thailandensis, P. noenieputensis, P. aegyptia* [90, 117]
*H. mexicana*		*P. luminescens* subsp. *mexicana* [124]
*H. noenieputensis*		*P. noenieputensis* [117]
*H. taysearae*		“P. sonorensis"* [167]
*H. atacamensis*		*P. antumapuensis, P. khanii* subsp. *guanajuatensis* [124, 126]
*H. downesi*	megidis	*P. cinerea, P. temperata* [117]
*H. marelatus*		*P. tasmanensis* [117]
*H. megidis*		*P. cinerea, P. temperata* [117]
*H. safricana*		*P. laumondii* subsp. *laumondii* [168]
*H. zealandica*		*P. tasmaniensis, P. heterorhabditis* subsp. *heterorhabditis, P. temperata* [117]
*H. egyptii*		Undescribed
*H. hambletoni*		Undescribed
*Heterorhabditis* sp.		*P. hainanensis* [125]
*Heterorhabditis* sp.		*P. hindustanensis* [169]
*Heterorhabditis* sp.		*P. heterorhabditis* subsp. *aluminescens* [125]
Undescribed		*P. australis* subsp. *australis* [125]

*This species was initially described as “*P. luminescens* subsp. *sonorensis*”, but its name was not validated. All the taxonomic changes proposed after its description, and our own analyses, show that it should be reclassified as “*P. sonorensis*”, but its name should be validated first by the original isolators

## Coevolution

Mutualistic microbial symbionts are often hypothesized to have undergone coevolution with their hosts, which can eventually lead to parallel speciation or co-speciation in both partners [170]. *Steinernema* species have a specific one-to-one relationship with *Xenorhabdus* spp. That is, one species of *Steinernema* may only be associated with one species of *Xenorhabdus* (Table 1; Fig. 3). However, certain promiscuous *Xenorhabdus* species can be hosted by several *Steinernema* species [171]. A single exception could be *S. sangi*, which has been reported to be associated both with *X. vietnamensis* [95] and *X. thuongxuanensis* [99]. It remains to be determined if this can also be explained by a misidentification because in none of the two studies, the nematode identification procedure is described in detail, however in both cases the authors claim that both *X. vietnamensis* [95] and *X. thuongxuanensis* (Phan, pers. comm.) were isolated from the type strain of *S. sangi*. The association of *S. sangi* with *X. vietnamensis* was further documented by molecular data for both the nematode and bacterium by Lalramnghaki et al. [172]. *Heterorhabditis* species associate with more *Photorhabdus* species [95] even within single populations [173] (Table 2; Fig. 4).

Several cophylogenetic studies on entomopathogenic nematodes and their bacterial symbionts have been performed in the past decades. Regarding the *Heterorhabditis–Photorhabdus* complex, Maneesakorn et al. [174], for instance, showed that phylogenies of nematodes and bacteria are consistent with a global co-speciation pattern, even though there are some mismatches between the two phylogenies in the case of *H. bacteriophora* and *H. georgiana* and their respective *Photorhabdus* symbionts. Given that the current number of species has increased dramatically since then, we synthesized the published data on *Photorhabdus–Heterorhabditis* associations up to the date (Table 2; Fig. 4). We observe that, although the different *Photorhabdus* species and subspecies are hosted by several *Heterorhabditis* species, there is a high degree of host specificity. Only four *Photorhabdus* taxa (*P. cinerea*, *P. laumondii* subsp. *laumondii, P. luminescens* subsp. *luminescens*, and *P. luminescens* subsp. *mexicana*) have been documented to be hosted by nematodes belonging to two different *Heterorhabditis* clades (“*bacteriophora*” and *“indica*”). The majority of heterorhabditid species have been observed to host only one and in few instances two *Photorhabdus* species/subspecies. However, *H. indica* and *H. bacteriophora* exhibit a higher degree of “promiscuity”, as they associate with numerous *Photorhabdus* species/subspecies from various *Photorhabdus* clades. This increased promiscuity may result from the broader distribution of these species, making them more commonly isolated and studied, and thus providing more data on their associations with *Photorhabdus*. Alternatively, as demonstrated in *H. downesi*, associating with different symbionts allows nematodes to expand their ecological niche [173]. The heightened promiscuity of species with the broadest distribution among heterorhabditid nematodes could therefore be an adaptation to colonize various habitats worldwide. Excluding these two “promiscuous” species, the species from the “indica” clade are generally associated with the most derived *Photorhabdus* clades (the “*P. aballayi*” and the “*P. noenieputensis*” clades); the nematodes from the “megidis” clade are found in association with more ancestral *Photorhabdus* species, such as *P. cinerea* and *P. tasmaniensis*, but in a few cases with transitional species such as *P. laumondii* subsp. *laumondii*; and species from the “bacteriophora” clade are associated with bacteria from a single *Photorhabdus* clade, the transitional clade “*P. laumondii*” (Fig. 4).

In the *Steinernema* and *Xenorhabdus* complex, the first study addressing co-speciation and the only study focused on the whole Steinernematidae family and all *Xenorhabdus* species found no evidence for co-speciation [163]. Instead, it revealed 12 co-speciation events and at least 17 host switches among the 30 *Steinernema*–*Xenorhabdus* pairs sampled [163]. Later studies documented switches of symbionts between nematodes of distantly related clades [149, 150] suggesting that switches may be frequent in the *Steinernema* and *Xenorhabdus* complex.

Generally, co-evolution is more easily documented in phylogenetic investigations of closely related species and intraspecific lineages [175–178]. In EPNs, Murfin et al. [179] sequenced genomes of nine *X. bovienii* strains and identified cocladogenesis between *Steinernema feltiae* nematode hosts and their corresponding *X. bovienii* symbiont strains, indicating potential specificity within the association. Recently, co-phylogenetic analysis revealed a remarkable congruence between phylogenies of the nematodes from “*bicornutum*” and ”*carpocapsae”* groups [144] and “*feltiae*” group [101] and their *Xenorhabdus* spp. symbionts.

The summary of the current data on *Steinernema* – *Xenorhabdus* associations (Table 1; Fig. 3) shows that some steinernematid clades are associated with a single *Xenorhabdus* species (for instance, nematodes of the “*affine*” clade with *X. bovienii*; nematodes of the “*monticola*” clade with *X. hominickii*) or with different, closely related *Xenorhabdus* species (“*kushidai*”, “*bicornutum*” and “*cameroonense*” clades), suggesting a potential co-evolutionary history. Nematode species from some clades, on the other hand, associate with more diverse, unrelated *Xenorhabdus* species (e.g., “*carpocapsae*” and “*feltiae*” clades). Similarly, bacteria from certain *Xenorhabdus* clades exclusively associate with nematodes from specific clades, such as the most basal *Xenorhabdus* clade and nematodes from the “*bicornutum*” and “*carpocapsae*” clades, while bacteria from other clades form association with more diverse and unrelated nematodes. Only four *Xenorhabdus* species establish association with nematode species belonging to different steinernematid clades (*X. bovienii* subsp. *bovienii*, *X. hominickii*, *X. khoisanae*, and *X. griffiniae*). Our interpretation of the available evidence is that some steinernematid lineages may have undergone co-speciation with their bacterial symbionts. This fact suggests that the specificity of the *Steinernema* spp. / *Xenorhabdus* spp. pairs might differ in different lineages. However the analyses of the coevolutionary history of *Steinernema* and *Xenorhabdus* is complicated by the fact that the identity of the bacterial symbiont is unknown in more than one-third of species, as well as due to a poor understanding of the relationships among *Steinernema* superclades.

## Conclusions

At present there are 113 species of *Steinernema* and 21 species of *Heterorhabditis*, and their delimitation is based mainly on sequence divergence. As the traditionally used genetic markers, the ITS and LSU regions of the rDNA lack the variability to distinguish closely related species, transitioning to multilocus molecular characterization will be necessary for future EPN systematics and species descriptions. In the phylogenetic reconstructions of EPNs, the ITS region of the rDNA proved to be the most powerful tool, enabling a division of both families into well-supported main clades. However, the relationships within clades, and in the case of steinernematids, also among clades, are not well resolved, and there is a need for additional genetic markers.

Both genera of symbiotic bacteria contain a similar number of species and subspecies with 32 *Xenorhabdus* and 30 *Photorhabdus* taxa. However, there are probably a higher number of undescribed *Xenorhabdus* species, as symbiont identity is unknown in more than one-third of steinernematid nematodes. In the last few years, the use of core genome sequences became the norm to describe novel species of *Xenorhabdus* and *Photorhabdus*. The same dataset is used for well-supported phylogenetic reconstructions.

The overview of *Heterorhabditis* – *Photorhabdus* associations and phylogenies confirms a high degree of host specificity, as heterorhabditids from particular clades tend to form association with the bacteria from specific *Photorhabdus* clades. However, numerous switches occurred during their co-evolutionary history. The high promiscuity of *H. indica* and *H. bacteriophora* could be an artefact of these widespread species being often isolated and studied. We hypothesize that, alternatively, the heightened promiscuity could be an adaptation to colonize various habitats worldwide.

In *Steinernema* –*Xenorhabdus* complex, some lineages may have undergone co-speciation, and it seems that the specificity of the *Steinernema* spp. / *Xenorhabdus* spp. pairs may differ in different lineages. However for a better understanding, more data on *Steinernema*–*Xenorhabdus* diversity and better tools for phylogenetic reconstruction of steinernematid nematodes are necessary.

### Electronic supplementary material

Below is the link to the electronic supplementary material.

Supplementary Material 1

## Data Availability

All data generated or analyzed during this study are included in this published article.
