# Supplementary material for: Systematics and phylogeny of the entomopathogenic nematobacterial complexes Steinernema–Xenorhabdus and Heterorhabditis–Photorhabdus
Source: Zoological Lett. 2024 Jul 17;10:13. doi: 10.1186/s40851-024-00235-y (PMC11256433; doi:10.1186/s40851-024-00235-y)
Supplement: Supplementary file 1 — Supplementary Material 1 [file 40851_2024_235_MOESM1_ESM.docx]

**Table S1**. National Center for Biotechnology Information (NCBI) accession numbers of the *Photorhabdus* genome sequences used in this study.

| *P. aballayi* APURE^T^ | JAPFCD01 |
| --- | --- |
| *P. aegyptia* BA1^T^ | JFGV01 |
| *P. akhurstii* subsp. *akhurstii* DSM 15138^T^ | RCWE01 |
| *P. akhurstii* subsp*. bharatensis* H3^T^ | PUWU01 |
| *P. antumapuensis* sp. nov. UCH-936^T^ | JAHZMK01 |
| *P. australis* subsp*. thailandensis* PB68.1^T^ | LOMY01 |
| *P. australis* subsp*. australis* DSM 17609^T^ | JONO01 |
| *P. asymbiotica* ATCC 43949^T^ | RBLJ01 |
| *P. bodei* LJ24-63^T^ | NSCM01 |
| *P. caribbeanensis* DSM 22391^T^ | RCWB01 |
| *P. cinerea* DSM 19724^T^ | PUJW01 |
| *P. hainanensis* DSM 22397^T^ | RCWD01 |
| *P. heterorhabditis* subsp*. aluminescens* Q614^T^ | JABBCS01 |
| *P. heterorhabditis* subsp*. heterorhabditis* SF41^T^ | RCWA01 |
| *P. hindustanensis* H1^T^ | PUWT01 |
| *P. kayaii* DSM 15194^T^ | JAJAFZ01 |
| *P. khanii* subsp. *khanii* DSM 3369^T^ | AYSJ01 |
| *P. khanii* subsp. g*uanajuatensis* MEX20-17^T^ | PUJY01 |
| *P. kleinii* DSM 23513^T^ | JAJAFY01 |
| *P. laumondii* subsp. *clarkei* BOJ-47^T^ | NSCI01 |
| *P. laumondii* subsp. *laumondii* TT01^T^ | WSFH01 |
| *P. luminescens* subsp. *luminescens* ATCC 29999^T^ | FMWJ01 |
| *P. luminescens* subsp. *mexicana* MEX47-22^T^ | PUJX01 |
| *P. luminescens* subsp. *venezuelensis* JAR^T^ | JAPFFZ01 |
| *P. namnaonensis* PB45.5^T^ | LOIC01 |
| *P. noenieputensis* DSM 25462^T^ | RCWC01 |
| *P. stackebrandtii* DSM 23271^T^ | PUJV01 |
| *P. tasmaniensis* DSM 22387^T^ | PUJU01 |
| *P. temperata* DSM 14550^T^ | JAJAFX01 |
| *P. thracensis* DSM 15199^T^ | CP011104 |

**Table S2**. National Center for Biotechnology Information (NCBI) accession numbers of the *Xenorhabdus* genome sequences used in this study.

| *X. aichiensis* XENO-7^T^ | JAQRFO01 |
| --- | --- |
| *X. anantnagensis* XENO-2^T^ | JAQRFN01 |
| *X. beddingii* Q58^T^ | MUBK01 |
| *X. bakwenae* SF857^T^ | CP119194 |
| *X. bovienii* subsp. *bovienii* T228^T^ | JANAIF01 |
| *X. bovienii* subsp. *africana* XENO-1^T^ | JAMGSK01 |
| *X. budapestensis* DSM 16342^T^ | NIBS01 |
| *X. cabanillasii* USTX62^T^ | QTUB01 |
| *X. doucetiae* FRM16^T^ | FO704550 |
| *X. eapokensis* DL20^T^ | MKGQ01 |
| *X. ehlersii* DSM 16337^T^ | NIBT01 |
| *X. griffiniae* ID10^T^ | JAQRFM01 |
| *X. hominickii* KE01^T^ | NJAI01 |
| *X. indica* DSM 17382^T^ | NKHP01 |
| *X. innexi* DSM 16336^T^ | NIBU01 |
| *X. ishibashii* GDh7^T^ | NJAK01 |
| *X. japonica* DSM 16522^T^ | FOVO01 |
| *X. khoisanae* SF87^T^ | JAQRFL01 |
| *X. koppenhoeferi* USNJ01^T^ | FPBJ01 |
| *X. kozodoii* SaV^T^ | NJCX01 |
| *X. lircayensis* VLS^T^ | JACOII01 |
| *X. magdalenensis* IMI 397775^T^ | JAQRFK01 |
| *X. mauleonii* VC01^T^ | NITY01 |
| *X. miraniensis* Q1^T^ | NITZ01 |
| *X. nematophila* ATCC 19061^T^ | FN667742 |
| *X. poinarii* G6^T^ | FO704551 |
| *X. romanii* PR06-A^T^ | JAQRFJ01 |
| *X. stockiae* TH01^T^ | NJAJ01 |
| *X. szentirmaii* DSM 16338^T^ | NIBV01 |
| *X. thuongxuanensis* 30TX1^T^ | MKGR01 |
| *X. vietnamensis* VN01^T^ | MUBJ01 |
| *X. yunnanensis* XENO-10^T^ | JAQRFI01 |
